# Specific Erosion Resistance Behaviour of Cold Forged and Angular Extruded Aluminium

**DOI:** 10.3390/ma17164070

**Published:** 2024-08-16

**Authors:** Zdenka Keran, Suzana Jakovljević, Biserka Runje, Igor Ciganović, Danko Ćorić

**Affiliations:** 1 Department of Technology, Faculty of Mechanical Engineering and Naval Architecture, University of Zagreb, Ivana Lučića 5, 10000 Zagreb, Croatia; zdenka.keran@fsb.unizg.hr (Z.K.); igor.ciganovic@fsb.unizg.hr (I.C.); 2 Department of Materials, Faculty of Mechanical Engineering and Naval Architecture, University of Zagreb, Ivana Lučića 5, 10000 Zagreb, Croatia; suzana.jakovljevic@fsb.unizg.hr; 3 Department of Quality, Faculty of Mechanical Engineering and Naval Architecture, University of Zagreb, Ivana Lučića 5, 10000 Zagreb, Croatia; biserka.runje@fsb.unizg.hr

**Keywords:** erosion wear resistance, forging, ECAP, statistical analysis

## Abstract

Aluminium finds wide application in mechanical engineering due to its low density and corrosion resistance. In this research, aluminium was subjected to two different metal forming technologies—cold forging (upsetting) and equal channel angular pressing (ECAP)—to obtain improvement in its exploitation properties. Parallel to changing mechanical properties by using these two processes, there was a change in the microstructure of the material. The resulting microstructures were examined using an optical microscope. A different treated aluminium was subjected to erosion wear in various time intervals. Wear testing was conducted for two different impingement angles causing abrasive wear and impact wear. The erosion mechanisms were examined by scanning electron microscopy. These results showed that there is no statistically significant difference in erosion wear for different states at the same impingement angle. However, the difference is noticeable at different wear angles. The significance of the difference in wear of the samples treated with the forging and ECAP techniques was validated by statistical analysis with tests of different sensitivities. The results of the *t*-test showed that ECAPed samples present a statistically significant difference in the loss of mass due to variations in erosion angle during the 30, 45, and 60 min wearing. A substantial difference in the change in sample mass is also visible for the forged state worn for 60 min.

## 1. Introduction

The mechanisms of cold plastic deformation that improve the mechanical properties of materials have long been known and widely researched. Such deformation process—open die cold forging—achieves the deformation of the grain in the direction of material flow, but it does not significantly affect the size of the crystal grain. More recently, newer metal forming processes, which increase the impact on the microstructure and mechanical properties of materials, have been studied. Among the most significant metal forming processes that affect the mechanical properties of light metals is the equal channel angular pressing (ECAP) technique, which comes to the fore in the research on the impact of significant plastic deformation (SPD) [1,2,3,4,5]. ECAP affects both the size of the grain and its deformation in the direction of the material flow. The focus of research in this area continues towards additional influential parameters on the results obtained by the ECAP procedure. In the first place, this refers to the ECAP in a hot state, which results in an even finer grain structure of the material [6]. It is followed by research papers on the influence of subsequent heat treatments on the final properties of materials pretreated by the ECAP process for aluminium alloys [7] and titanium alloys [8,9]. The mechanical properties after these treatments and the obtained ultrafine grain size (UFG) remain stable even at elevated temperatures, which, for Al–Zn–Mg alloys, is 473 K [10]. The research results that combine the wear investigation of the previously achieved UFG microstructure with the forging process showed the transformation of wear mechanisms with the annealing temperature after plastic deformation [11].

Aluminium alloys are widely used in aerospace, automotive, and structural applications due to their favourable strength-to-weight ratio. Enhancing wear resistance is one of the critical features for extending the lifespan of aluminium components. Understanding the wear behaviour of aluminium processed through different cold forming processes can inform material selection and processing techniques in industrial applications. Investigations of the mechanisms and consequences of wear of light metals that are cold-formed by forging or pass through severe plastic deformation by ECAP process have been carried out more recently to improve the possibility of using the influence of cold deformation to increase usability [12,13]. The study of the effects of ECAP processing routes on the wear property of Al–Al3Ti alloys showed that route A results in an anisotropic microstructure, unlike the BC route, which results in a homogenous microstructure. Whichever route is used, the material will not show anisotropic properties due to the wear test [14]. However, the research results are often conflicting because numerous factors can influence the correlation of wear properties and material hardness. Scientific papers report a decreasing wear resistance when an aluminium alloy is treated by the ECAP process, and significant grain refinement is obtained [15,16]. The possible cause of these results is the lack of a strain hardening capability. Comparable results are reported for copper alloy [17]. Opposite results were obtained for the Al–Mg–Zn alloy, where the wear rate was decreased with the increased number of ECAP passes. The microstructure refinement and the improvement of mechanical properties led to the transformation of the wear mechanism [18]. Additionally, the research of the dry sliding wear behaviour of the Al–Si–Mg alloy showed a lower wear rate of ECAPed samples [19]. Research results that combine the wear investigation of the previously achieved UFG microstructure with the forging process showed the transformation of wear mechanisms with the annealing temperature after plastic deformation [11].

The literature also records research on the influence of cold forging on the wear resistance of materials. Research on the influence of cold forging on the wear behaviour of spray-formed Al–Si alloys reports that samples with 75% thickness reduction showed higher wear resistance compared with those in the initial state [20]. Another research, on the wear resistance of cold-forged high carbon steel, showed that the wear rate of high carbon steel decreased with an increase in hardness obtained by the cold forging process [21].

Using molecular dynamics modelling, the general influence of cold deformation on the wear resistance of the material is presented. According to this research, the correlation of the ratio of hardness to the elastic modulus (H/E) influences the wear resistance of metallic materials. A higher wear resistance is caused by a larger H/E ratio. However, in the H/E ratio caused by cold plastic deformation, the wear resistance is not higher, because plastic deformation causes crystalline defects (dislocations). These defects lead to deteriorated crystalline integrity [22].

One of the wear types is erosive wear in which material degradation takes place due to the impact of external particles on the surface of the material. This type of wear, slurry erosion, often occurs in aluminium parts, especially pipes through which a solid or liquid medium containing eroding particles flows. Slurry erosion is a complex, time-dependent phenomenon that depends on many different parameters, such as eroded material properties (microstructure, ductility), erosive particle characteristics (impact angle of eroding particle, velocity of impact, material of erodent, size, and shape of eroding particle), and operating conditions [23,24,25,26,27,28,29]. According to the mentioned influential erosion parameters, the mechanisms of erosion wear that occur on the surface of the material are cutting and deformation [30,31,32].

At small angles of particle flow towards the surface, an abrasive erosion occurs, and when the angle of particle flow increases to 90°, it takes an impact erosion character [33,34].

The ductility of the target surface influences the total erosion [35,36,37,38,39]. Ductile materials achieve the lowest wear resistance at a low impingement angle. On the contrary, with brittle materials, the wear rate increases with an increase in erosion angle and is maximum at a higher erosion angle [33]. The erosion of ductile materials at a low impact angle involves material removal by cutting/ploughing, while under a normal impact angle, it causes plastic deformation and creates a crater with extruded lips. The erosion of brittle materials occurs through the formation of a network of subsurface cracks [40].

This paper investigates the erosion wear resistance of unalloyed aluminium processed by two cold forming processes: open die forging and ECAP technique. This study was performed in the context of improving the usability of aluminium by influencing its hardness by cold plastic deformation in these two processes. A comparison of the effects of open die forging and ECAP on wear resistance has not been extensively made. The question of whether there is a statistically significant difference between the wear of forged and ECAPed material arises since the forging process is simpler and cheaper in its implementation. It is especially important to make a statistical evaluation of the comparison of the wear results, which is performed in this paper. The intention of this research is to provide recommendations for the use of the appropriate plastic deformation in order to achieve a satisfactory level of resistance to erosion wear. The behaviour of cold-formed aluminium by open die forging and the ECAP process has been studied for two different wear mechanisms—abrasive erosion and impact erosion—on which there are no insufficient recent data. This study uses a combination of wear tests and statistical analysis to verify experimental results.

## 2. Materials and Methods

All tests were performed on samples that are made of commercially pure aluminium Al 1050 (99.5) (thyssenkrupp Materials, Preston, UK) with the composition shown in Table 1. A chemical analysis was conducted using an optical emission spectrometer, GDS 850A (Leco, Saint Joseph, MI, USA).

Samples were classified according to their state depending on the treatment method. The first state represents hot-rolled aluminium, which is also the supply state of the material. This is a control sample since it has suffered no cold deformation. The second state is presented by cold open die forging with 60% deformation. A drop hammer was used to forge the samples—the weight of hammer was 85 kg. The drop hammer was built at the Faculty of Mechanical Engineering and Naval Architecture, University of Zagreb, and the forging was conducted at the same faculty in the Laboratory for Metal Forming. The lifting height of the hammer was 1.2 m. It is possible to control the amount of plastic deformation of the material during cold open die forging by selecting the compression height of the sample. By controlling the height of the hammer’s fall, the energy that causes the plastic deformation of the material is also controlled. Given the expected final height of the sample, it is necessary to select the appropriate height of the hammer’s fall. Forging is a metal forming process that has been traditionally used not only to change the product shape, but also to improve mechanical properties and change the microstructure of the material. The third state was treated with ECAP in one pass. ECAP is a process that has been developed in recent times and is primarily intended to refine grain size and achieve better mechanical properties rather than changing the shape of a product. The amount of plastic deformation of the material treated with ECAP is defined by the angle of the tool die. The largest plastic deformation (in one ECAP pass) is achieved in the case of a die angle of 90° degrees, which is the case in this research.

**Figure 1:**
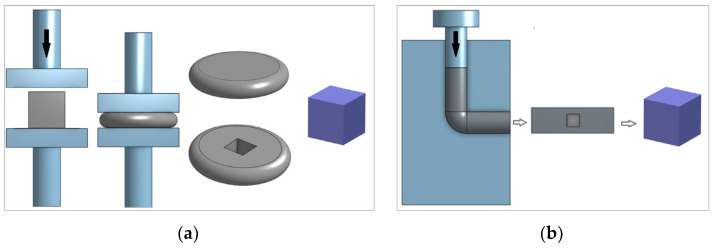
The schemes of the plastic deformation process and sampling: (**a**) open die forging; (**b**) ECAP process.

After plastic deformation, the samples were cut out into cubes with a 15 mm long edge. The schemes of the forging and ECAP processes and the position of taking samples are shown in Figure 1.

The process of preparing samples for microstructural analysis was as follows:

- Crosscutting of samples performed on a Struers ACCUTOM-2 precision cutter with an Al_2_O_3_ cutting plate (Struers, Champigny-sur-Marne, France);
- Fixing in polymer compound;
- Grinding with sandpaper of different grits: 220, 320, 500, and 800;
- Pre-polishing with DPMol diamond paste with a particle size of 3 μ
- Final polishing—OP Chem suspension with a particle size of 1/4 μ
- Washing—ethyl alcohol;
- Drying in a stream of warm air.

Samples prepared in this way are etched by the so-called Keller’s reagent for 30 s. The composition of Keller’s reagent is 2 mL HF, 3 mL HCl, 5 mL HNO_3_, and 190 mL distilled water. Microstructural analysis was performed using an Inverted Metallurgical Microscope Olympus GX51 (Olympus, Evident, Berlin, Germany).

The metallographic preparation of a sample was conducted by mounting, grinding, polishing, and etching. Grinding on the device Phoenix Alpha (Buehler, Lake Bluff, IL, USA) was performed in four steps using the following SiC sandpapers: P500, P800, P1200, and P4000. The rotation speed of the grinding plate was 300 rpm, and the samples were cooled with water to prevent overheating and microstructure transformations. The grinding at each stage lasted 0.5 min using a force of 25 N. After that, a two-step polishing process was followed on the same device. A cloth with diamond paste was applied as a polishing base, and DiaPro Mol R3 and UP-U as a lubricant. In the first step of polishing for 4 min, diamond paste with 3 µm abrasive grain was used, and then polishing liquid with an abrasive size of 0.03 µm was applied for 3 min. During polishing, the plate rotated at 150 rpm, and the force was 25 N (first step) and 15 N (second step). Etching was carried out in a solution with the following composition: 90 mL water, 15 mL hydrochloric acid, and 10 mL hydrofluoric acid.

The sample hardness of different states was measured after microstructural analysis. A hardness test by the Vickers method was performed on an Indentec hardness tester (ZwickRoell, Ulm, Germany), with an indentation load of 9.81 N (HV1). Ten hardness measurements were made for each sample state, and the mean value and standard deviation were calculated.

**Figure 2:**
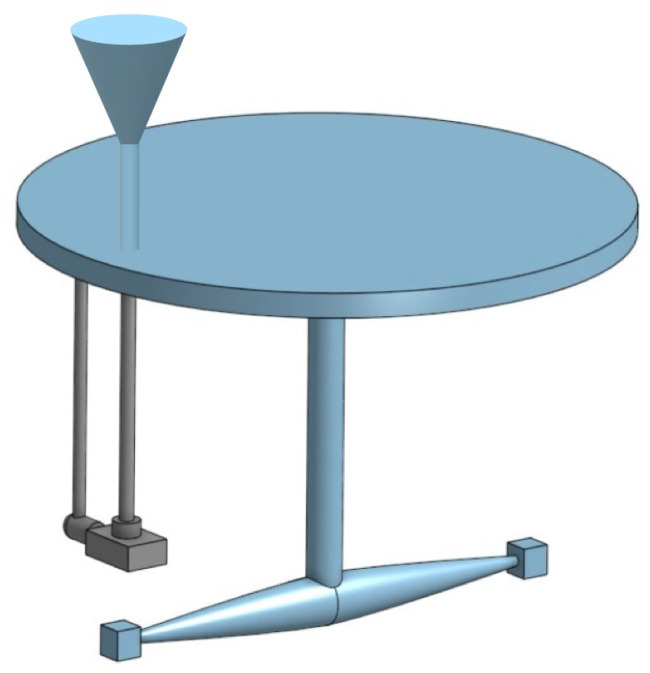
The scheme of the experimental method—whirling arm tester.

The wear testing was performed using an erosion wear test device—the whirling arm tester (FAMENA, Zagreb, Croatia), shown in Figure 2, which uses the rotation of samples that hit the stream of erosion particles. Erosion particles fall freely under the influence of gravitational force. Samples are placed in two holders fastened with screws. By regulating the position of the sample surface according to the falling particles, it is possible to conduct an examination at different impingement angles. In this study, the samples were mounted in such a way that the erosion particles hit at angles of 30° and 90°, respectively. In this way, the tests were conducted for two different erosion wear mechanisms: abrasive and impact erosion. Preliminary measurements showed that there was no difference in the mass loss regarding the orientation of the sample according to the direction of flow of the material. This applies to both the cold forging process and the ECAP process. It is necessary to notice that the preliminary measurements coincide with the previously described literature sources [14].

The rotational speed of the samples is 1440 rpm, which gives 24.3 m/s as the speed of collision of the sample surface with the particles. The erosion particles flow through a nozzle with a diameter of 5 mm. Quartz sand Ottawa AFS 50/70 was used as an erodent. The test time was set at 15, 30, 45 and 60 min. The intensity of wear is defined by the change in the mass of the sample. Therefore, the sample mass was measured before and after each wear test. To determine the mass loss (Δ*m*), a precision balance with a resolution of 0.0001 g (type B5C 1000, Mettler Toledo, Zurich, Switzerland) was used. The mass loss (Δ*m*) was measured as

(1) $$∆m=m_{0}-m_{t}$$

where *m*_0_ is initial mass and *m_t_* is mass after wear time (15, 30, 45, 60 min).

Wear tests were performed for each wear time (15, 30, 45, 60 min) and each type of sample state with twelve repetitions. The mass measurement of each sample was performed three times under repeatability conditions. Repeatability conditions imply measurements conducted within a short interval of time by a single operator using the same equipment. The standard deviation of repeatability *s_r_* was determined to be *s_r_* = 0.000048 g.

In accordance with the international standard ISO 5725-2:2019, it may be expected, with a probability of 95%, that the absolute difference between two test results obtained under repeatability conditions will be less than or equal to the repeatability limit, which amounts to 0.0002 g [41].

Samples are marked in Table 2 as follows:

- Sample state: supply state (S), forged (F), ECAPed (E);
- Impact angle: 30° (A), 90° (B);
- Wear duration: 15 min (15), 30 min (30), 45 min (45), 60 min (60).

Scanning electron microscopy (SEM) was performed after erosion tests. The analysis of the worn surface of the samples was carried out using TESCAN VEGA 5136 MM (TESCAN Brno, Brno, Czech Republic).

## 3. Results and Discussion

**Figure 3:**
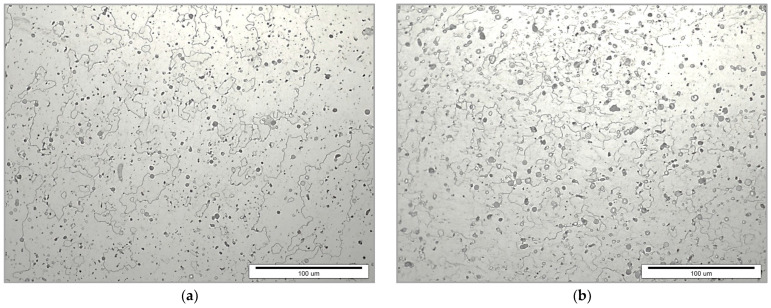

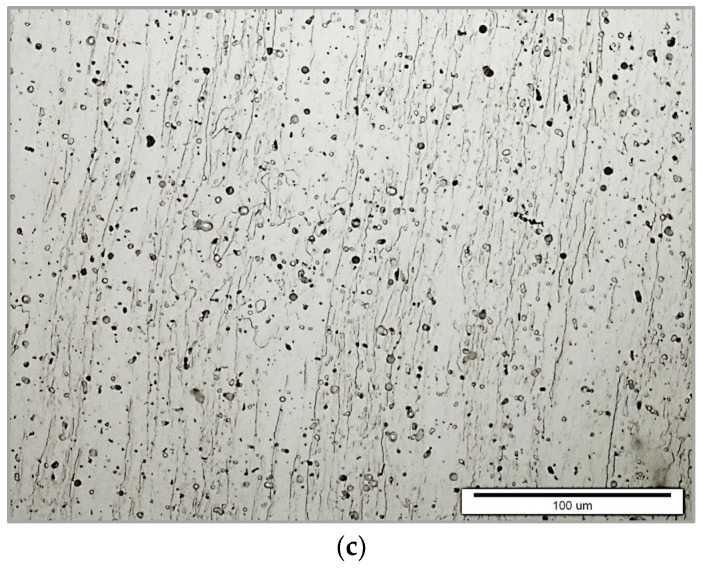
Microstructures of different plastic-deformed states: (**a**) hot-rolled, (**b**) cold-forged, and (**c**) ECAPed.

Different plastic deformation processes resulted in various aluminium microstructures, as show in Figure 3. The microstructure of the supply state, i.e., hot-rolled aluminium, is homogeneous due to forming at high temperatures. The microstructure after cold forging characterizes hardly visible grain elongation, while the microstructure achieved by ECAP shows the elongated grains in the direction of material flow. By observing the direction of grain elongation, it is possible to determine the direction of material flow. The elongation of the grain always follows the direction of material flow. Greater plastic deformation causes greater material flow and, consequently, greater elongation of the grain.

The effect of cold plastic deformation on the mechanical properties of the material resulted in an increase in hardness concerning the supply state. The forged state indicates a smaller increase in hardness, while the ECAP state results in a greater increase in hardness of almost 40% compared with the initial state. The results of hardness measurement as a mean value of ten measurements and standard deviation are shown in Table 3.

**Figure 4:**
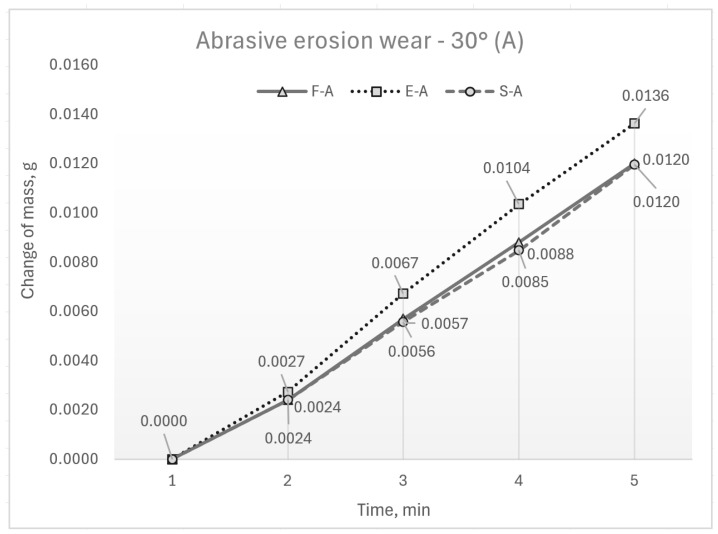
Mass loss at 30° impingement angle.

**Figure 5:**
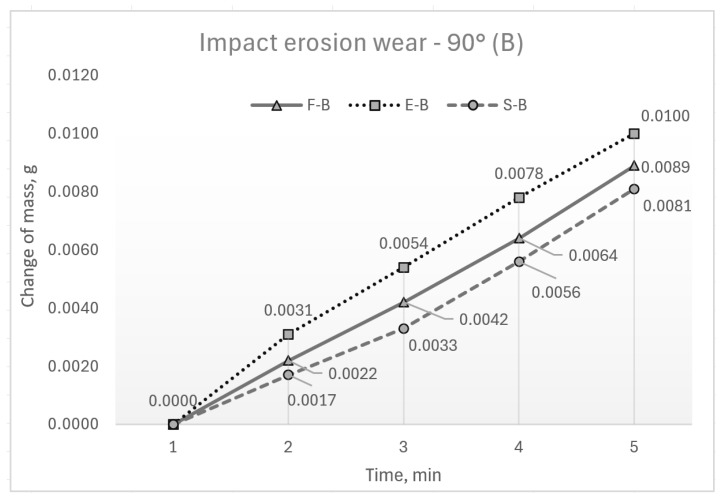
Mass loss at 90° impingement angle.

The results of the wear testing are presented through the change in the sample mass. The mean values of mass loss are shown in graphs in Figure 4 and Figure 5. These figures show differences in wear concerning the state of the samples induced by different processing treatments and with respect to the slope of the sample surface to the abrasive flow. According to the reported mean values of test results, wear grows as aluminium hardness increases. Therefore, the lower wear, regardless of the impingement angle, is visible in samples in the supply state with the lowest hardness. Some higher wear is visible in forged samples, and the highest wear is shown by the hardest ECAP samples. More significant differences are evident in impact erosion wear (90°). It is also evident that the slopes of the curves describing erosion wear are very close in the interval of 30 to 60 min. This indicates the same dynamics of wear in this time interval.

When the obtained results are compared with the results of previous scientific research, it is possible to observe some correspondence with the results stated in the literature. Similar results of the wear of ECAPed material are described in [15,16,18]. Results of wear for forged material are described in [22]. However, these results are not described for the same material, and there is no comparison of the wear of the same material in the case of cold plastic deformation by forging and ECAP.

The presented results were additionally subjected to statistical analysis to be able to draw valid conclusions about the influence of the considered factors (sample state, impact angle, test duration) on the erosive wear of the examined aluminium.

## 4. SEM Analysis

**Figure 6:**
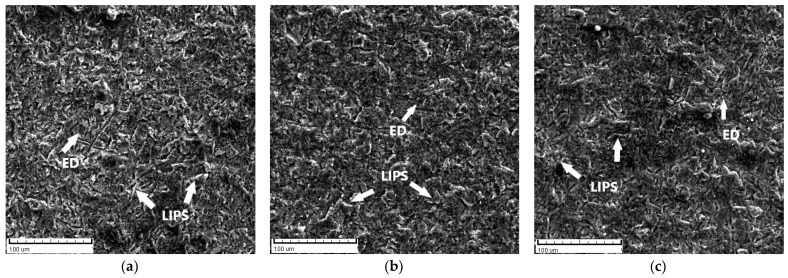
Sample surfaces eroded at an impingement angle of 30° for (**a**) supply state, (**b**) forged, and (**c**) ECAPed.

**Figure 7:**
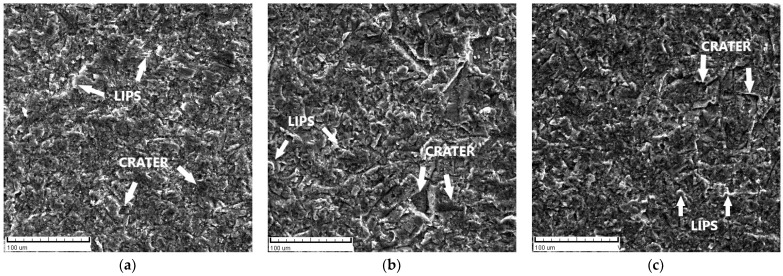
Sample surfaces eroded at an impingement angle of 90° for (**a**) supply state, (**b**) forged, and (**c**) ECAPed.

Scanning electron microscopy (SEM) was performed by using TESCAN VEGA 5136 MM to analyse the surfaces of samples after the erosion test. In the present study, SEM analyses were conducted on all samples (supply state, forged F, and ECAPed) after they were treated for 60 min at two impact angles, 30° and 90°. Eroded surfaces of samples are shown in Figure 6 and Figure 7. The ED designation in Figure 6 stands for Erosion Direction.

**Figure 8:**
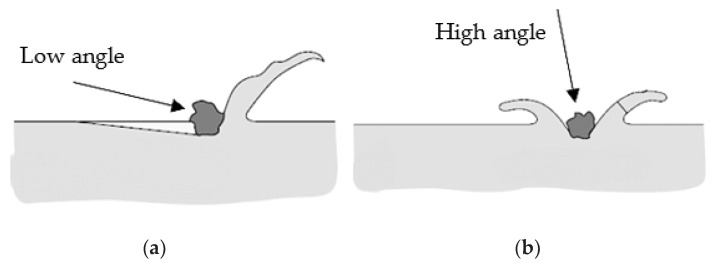
Various erosive wear mechanisms: (**a**) abrasive erosion and (**b**) impact erosion [42].

SEM micrographs for all samples, supply state, forged, and ECAPed, at a 30° impact angle are presented in Figure 6a–c. It is shown that the wear is contributed by microcutting and microploughing, which are well-known mechanisms of erosion of ductile materials at low impact angles [42,43]. At an oblique impact angle (30°), hard erodent particles (quartz sand) have a large horizontal component of velocity, which spreads the material in the particle flow direction. This displaces the material in the flow direction and forms the lips, as shown in Figure 8a.

The wear at a 90° angle is contributed by deformation wear and microcutting, as shown in Figure 7a–c. At this angle, erodent particles have a large vertical component of velocity, which causes plastic flow in soft surfaces and forms deeper craters and sharper lips, as shown in Figure 8b. Based on SEM, in Figure 6 and Figure 7, it is observed that the microcutting and the smeared craters (caused by microploughing) are dominant in all states of samples (supply state, forged, and ECAPed) at an oblique impact angle (30°). Furthermore, there is no significant difference in the worn surface topography of differently treated samples (supply state, forged, ECAPed) at the same impact angle.

## 5. Statistical Analysis

A statistical analysis of the results of sample mass loss due to erosion wear was conducted. The influence of time of wear and the influence of different metal forming technologies and different erosion angles on the change in the mass of the samples were analysed. The analysis of variance (ANOVA) procedure was applied. Additionally, multiple comparisons of the means of change in the mass were conducted using the Games–Howell Welch test and two-sample *t*-test.

**Figure 9:**
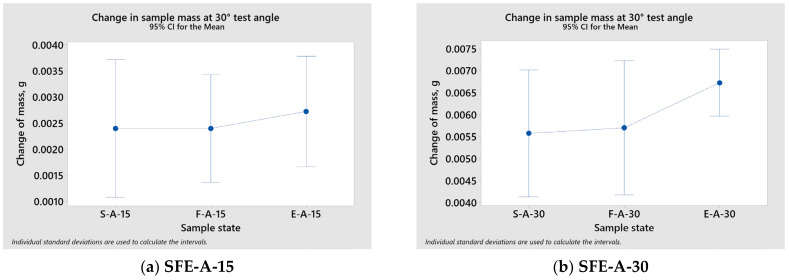

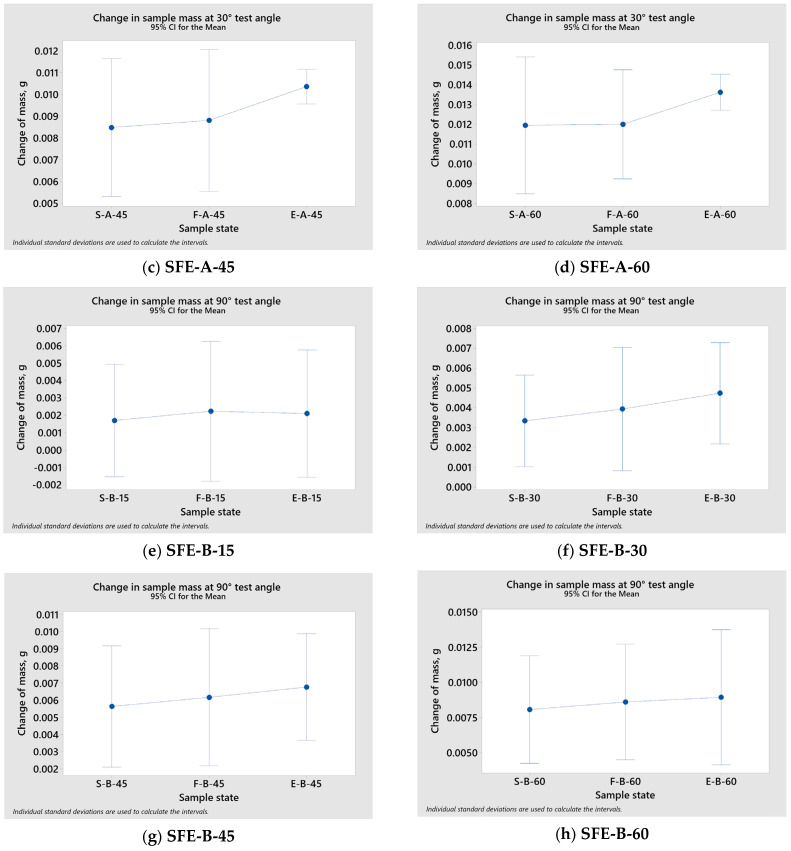
The interval plot of the mean and confidence interval for change in mass depending on the sample state (**a**–**h**).

Using the ANOVA method, the influence of the sample state (S—supply state, F—forged, E—ECAPed) on change in mass was analysed. The results of the ANOVA method are presented in Table 4. The interval plot of the mean and confidence interval for change in mass depending on the sample state is presented in Figure 9.

Using the ANOVA method, it can be determined that all *p*-values in Table 4 are greater than the significance level (alpha) of 0.05. The *p*-values represent sufficient evidence to support the conclusion that all of the means of the change in sample mass for different sample states are equal when the significance level (alpha) is set at 0.05.

**Figure 10:**
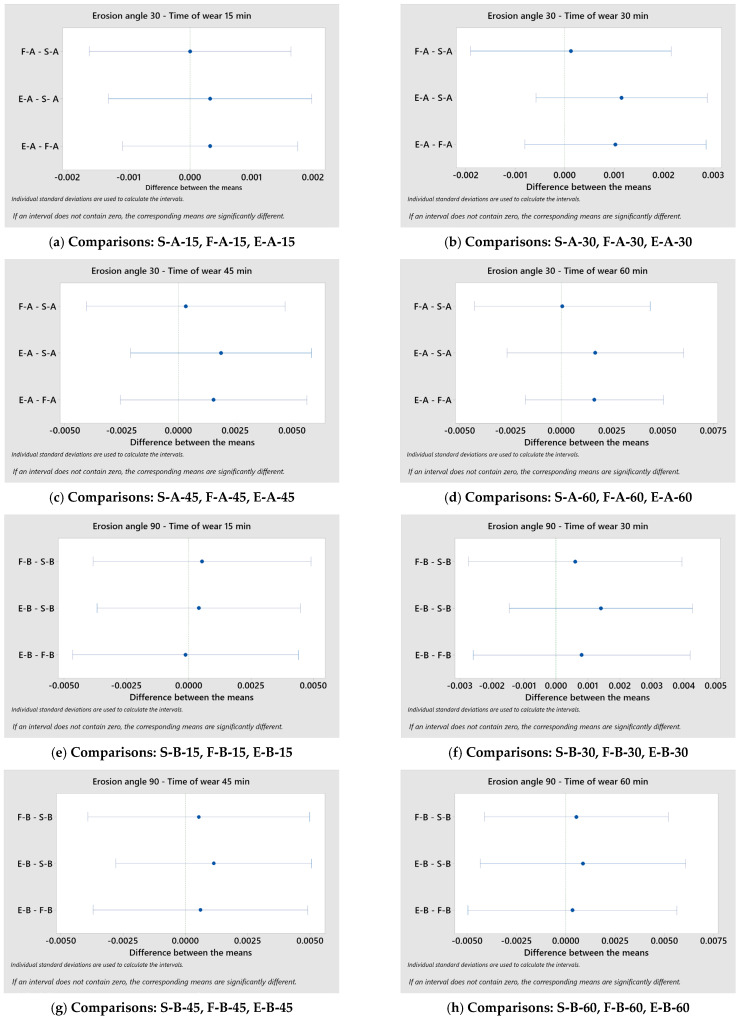
Results of the multiple comparisons Games–Howell Welch test (**a**–**h**).

In order to explore the differences in the loss of mass with respect to the sample state for different times of wear and different impingement angles, multiple comparison results were examined. The multiple comparisons Games–Howell Welch test was used (Figure 10).

In accordance with the Games–Howell Welch test, if the interval does not contain zero, the corresponding means are significantly different. It is evident that, for different impact angles, the sample means of change in mass, with respect to the sample state, are not significantly different when the alpha is set at 0.05.

**Figure 11:**
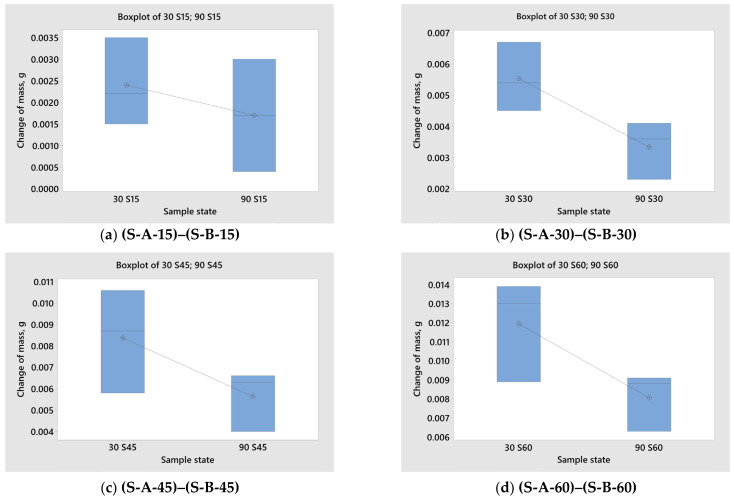

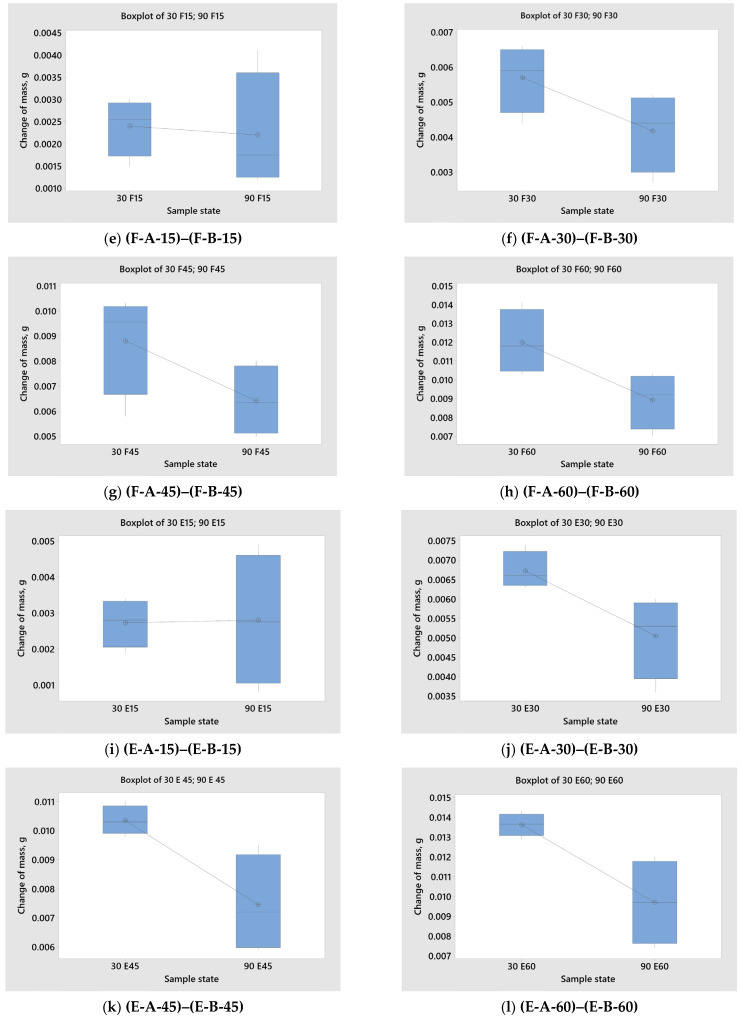
Box plots—the influence of the impingement angle on the change in mass (**a**–**l**).

Using the two-sample *t*-test, the influence of the impact angle on the change in mass was also analysed (Table 5). To compare the central tendency and variability of measurement results and to look for outliers, measurement results are graphically presented in box plots (pox and whisker plots) in Figure 11.

Based on the results of the *t*-test shown in Table 5 and the box and whisker plots in Figure 11, it follows that when applying the ECAP procedure, there is a statistically significant difference in the results (*p* < 0.05) of the change in sample mass due to the change in the erosion angle. A statistically significant difference in the change in sample mass was determined for 30, 45, and 60 min duration of wear. Additionally, a statistically significant difference in the loss of sample mass was determined for the forged state at 60 min wear time.

## 6. Conclusions

This study investigated the wear resistance in abrasive erosion and impact erosion cases of pure aluminium 1050 (99.5). Experimental samples were in three states—supply state (hot-rolled)—and in two states that suffered cold plastic deformation—open die forging and ECAP. The aim of this research was to compare the resistances to erosion wear of these three states to provide an adequate recommendation on the use of cold deformation processes (open die, forging, and ECAP) to improve the erosion wear resistance of unalloyed aluminium.

According to the reported mean values of the measurement results, wear increases with the increase in sample hardness. SEM analysis showed no significant difference in the surfaces of differently treated samples worn at the same impingement angle. However, there is a difference in topography for different impact angles.

Making conclusions about the significance of the difference in the wear of samples treated with different cold plastic deformation procedures (forging and ECAP) requires statistical analysis with tests of different sensitivities. The ANOVA method indicates that all the means of the change in sample mass for different sample states are equal when the significance level (alpha) is set at 0.05. In accordance with the Games-Howell Welch test, it can be concluded that, for different impingement angles, the loss of sample mass considering the sample state is not significantly different when the alpha is set at 0.05. Based on the results of the *t*-test, it can be concluded that ECAPed samples showed a statistically significant difference in the results of the change in mass due to variations in erosion angle for 30, 45, and 60 min wear durations. Additionally, a significant difference in the loss of sample mass was reported for the forged state worn for 60 min. The amount of abrasive erosion (impingement angle of 30°) was higher than that of impact erosion (impingement angle of 90°). The samples in the control condition (hot-rolled) do not show any statistically significant difference considering two erosion angles.

Due to all of the above, it is possible to make several recommendations. Since, in the cases of abrasive erosion wear, ECAPed samples showed the best results, in cases where unalloyed aluminium is exposed to abrasive erosion wear, it is recommended to treat it with ECAP. Since, in the case of short-term impact erosion wear, ECAPed and forged samples showed close results (statistically analysed), in cases of exposure to impact erosion wear, it is equivalent to using forged material, especially if the time of wear is short. If there are no exploitation requirements for the increased hardness of the material, it is possible to use the material in the supply (hot-rolled) state equivalently.

## Figures and Tables

**Table 1:** Chemical composition of examined aluminium.

Element, wt. %							
Fe	Si	Zn	Mn	Mg	Cu	Ti	Al
0.304	0.120	0.023	0.016	0.011	0.011	0.008	bal. *

* note: bal. is the abbreviation of balance.

**Table 2:** Matrix of samples.

	15 min	30 min	45 min	60 min
A	S-A-15	S-A-30	S-A-45	S-A-60
	F-A-15	F-A-30	F-A-45	F-A-60
	E-A-15	E-A-30	E-A-45	E-A-60
B	S-B-15	S-B-30	S-B-45	S-B-60
	F-B-15	F-B-30	F-B-45	F-B-60
	E-B-15	E-B-30	E-B-45	E-B-60

**Table 3:** Hardness values of various plastic-deformed states.

State	Hot-Rolled—Supply State	Cold-Forged	ECAPed
Hardness, HV1	41 ± 4	47 ± 6	57 ± 5

**Table 4:** ANOVA—the influence of the sample state on change in sample mass.

Time (min)	Erosion Angles	F-Value	*p*-Value
15	30° (A)	0.27	0.772
	90° (B)	0.10	0.908
30	30° (A)	3.21	0.121
	90° (B)	1.31	0.366
45	30° (A)	2.28	0.209
	90° (B)	0.46	0.659
60	30° (A)	2.18	0.215
	90° (B)	0.17	0.849

**Table 5:** Two-sample *t*-test—the influence of the impingement angle on the change in mass.

Sample State	Difference in Change in Mass	T-Value	*p*-Value
Supply state—S	(S-A-15)–(S-B-15)	0.74	0.516
	(S-A-30)–(S-B-30)	2.64	0.078
	(S-A-45)–(S-B-45)	1.69	0.190
	(S-A-60)–(S-B-60)	2.18	0.118
Forged—F	(F-A-15)–(F-B-15)	0.27	0.800
	(F-A-30)–(F-B-30)	2.06	0.095
	(F-A-45)–(F-B-45)	1.91	0.114
	(F-A-60)–(F-B-60)	2.69	0.044
ECAPed—E	(E-A-15)–(E-B-15)	0.08	0.944
	(E-A-30)–(E-B-30)	2.90	0.044
	(E-A-45)–(E-B-45)	3.26	0.047
	(E-A-60)–(E-B-60)	3.45	0.041

## Data Availability

The data presented in this study are available upon request from the corresponding author.
